# AMPK activation caused by reduced liver lactate metabolism protects against hepatic steatosis in MCT1 haploinsufficient mice

**DOI:** 10.1016/j.molmet.2017.10.005

**Published:** 2017-10-20

**Authors:** Lionel Carneiro, Mohamed Asrih, Cendrine Repond, Christine Sempoux, Jean-Christophe Stehle, Corinne Leloup, François R. Jornayvaz, Luc Pellerin

**Affiliations:** 1 Département de Physiologie, Université de Lausanne, 1005 Lausanne, Switzerland; 2 Service of Endocrinology, Diabetes and Metabolism, Centre Hospitalier Universitaire Vaudois, 1011 Lausanne, Switzerland; 3 UMR CNRS 6265 – INRA 1324 – AgroSup, Univ. Bourgogne Franche-Comté, Center for Taste and Feeding Behaviour, 21000 Dijon, France; 4 Pathologie Clinique, Institut Universitaire de Pathologie-IUP, 1011 Lausanne, Switzerland; 5 Mouse Pathology Facility UNIL-CHUV, 1066 Epalinges, Switzerland; 6 Centre de Résonance Magnétique des Systèmes Biologiques, UMR5536 CNRS, LabEx TRAIL-IBIO, Université de Bordeaux, Bordeaux Cedex 33760, France

**Keywords:** Liver, Obesity, NAFLD, Diabetes, Lactate, NAFLD, Non-Alcoholic Fatty Liver Disease, NASH, Non-Alcoholic SteatoHepatitis, ROS, Reactive Oxygen Species, ER Stress, Endoplasmic Reticulum stress, HFD, High Fat Diet, FA, Fatty Acids, TAG, TriAcylGlycerides, MCT1, Monocarboxylate Transporter isoform 1, SREBP1, Sterol Regulatory Element Binding Protein 1, AMPK, AMP-activated protein kinase, SD, Standard Diet, Rd, Glucose disappearance rate, HGP, Hepatic Glucose Production, GIR, Glucose Infusion Rate, LDHA, Lactate DeHydrogenase isoform A, LDHB, Lactate DeHydrogenase isoform B, a.u.c, Area Under the Curve, ACC, Acetyl-CoA Carboxylase, mTOR, mammalian Target Of Rapamycin, S6K, Ribosomal protein S6 kinase, PC, Pyruvate Carboxylase

## Abstract

**Objective:**

Hepatic steatosis is the first step leading to non-alcoholic fatty liver disease, which represents a major complication of obesity. Here, we show that MCT1 haploinsufficient mice resist to hepatic steatosis development when fed a high fat diet. They exhibit a reduced hepatic capacity to metabolize monocarboxylates such as lactate compared to wildtype mice.

**Methods:**

To understand how this resistance to steatosis develops, we used HFD fed wildtype mice with hepatic steatosis and MCT1 haploinsufficient mice to study hepatic metabolism.

**Results:**

AMPK is constitutively activated in the liver of MCT1 haploinsufficient mice, leading to an inactivation of SREBP1. Therefore, expression of key transcription factors for lipid metabolism, such as PPARα and γ, CHREB, or SREBP1 itself, as well as several enzymes including FAS and CPT1, was not upregulated in these mice when fed a high fat diet. It is proposed that reduced hepatic lactate metabolism is responsible for the protection against hepatic steatosis in MCT1 haploinsufficient mice via a constitutive activation of AMPK and repression of several major elements involved in hepatic lipid metabolism.

**Conclusion:**

Our results support a role of increased lactate uptake in hepatocytes during HFD that, in turn, induce a metabolic shift stimulating SREBP1 activity and lipid accumulation.

## Highlights

• Lactate uptake causes a shift in LDH isoform expression during HFD.
• LDH isoform shift favors a decrease in AMPK activity.
• Lactate uptake reduction in MCT1+/− mice blocks LDH isoform shift and leads to AMPK activation.
• SREBP activation is prevented in MCT1+/− mice due to AMPK activation.
• Decreased expression of SREBP targets involved in lipid metabolism protects against NAFLD.

## 1 Introduction

Obesity represents a worldwide health problem. Several studies have highlighted the involvement of the liver in the complications linked to obesity [1], [2], [3]. Hepatic steatosis is mainly characterized by fat accumulation in liver cells. Such excessive lipid accumulation can evolve into non-alcoholic steatohepatitis (NASH), cirrhosis, and, finally, hepatocellular carcinoma, three distinct stages of non-alcoholic fatty liver disease (NAFLD) [4]. Obesity is one of the most common causes of NAFLD [5]. However, the mechanisms leading to NAFLD remain incompletely understood even though several factors have been implicated, such as genetics, reactive oxygen species (ROS), endoplasmic reticulum stress (ER stress), inflammasome, and others [6], [7], [8], [9].

Animal models involving high fat diet (HFD)-induced hepatic steatosis have been extensively used to investigate this disorder [10]. Many studies have highlighted the role of circulating fatty acids (FA) and triacylglycerides (TAG) that tend to accumulate in the liver when their blood concentration increases. This accumulation alters mitochondrial function, causing oxidative stress, lipid peroxidation, and an inflammatory response that participate to insulin resistance development [11], [12]. Once NAFLD has developed, this negative loop leads to inflammation and sustained lipotoxic action, allowing the progression to NASH, cirrhosis, and hepatic carcinoma [13]. HFD models also unraveled a role of decreased fat oxidation in association with a rise in lipogenesis. These observations clearly emphasize the importance of lipid metabolism balance in hepatic steatosis development [14], [15].

In this regard, the recent development of a mouse model haploinsufficient for the monocarboxylate transporter isoform 1 (MCT1) which exhibits resistance to HFD-induced obesity and associated NAFLD is interesting for the understanding of the pathology onset [16]. MCT1 has the broadest substrate selectivity and tissue distribution among all members of the SLC16 protein family. MCT1 transports short-chain monocarboxylates such as lactate, pyruvate, and ketone bodies [17]. The role of these energy substrates and their transporters in energy homeostasis regulation remains unclear [18]. In the MCT1 haploinsufficient mouse model (MCT1+/−), a reduced upregulation of hepatic lipogenic genes was evidenced compared to the wildtype littermate following HFD. In particular, the expression of the sterol regulatory element binding protein 1 (SREBP1), a key lipogenic transcription factor, is lower in the liver of MCT1+/− mice [16]. Although this previous study revealed a central role of MCT1 in diet-induced obesity, it did not identify the mechanisms by which the partial MCT1 invalidation protects against NAFLD.

Recently, it was demonstrated that the fuel-sensing enzyme AMP-activated protein kinase (AMPK) inhibits SREBP1 activity [19]. Consequently, this inhibition contributes to the resistance to hepatic steatosis. Moreover, AMPK is a key factor that regulates several cellular processes including glucose uptake and lipid metabolism [20]. AMPK is rapidly activated when the AMP/ATP ratio increases. Once activated, AMPK promotes energy producing processes, while it inhibits energy consuming ones such as protein or fatty acid synthesis in liver [21]. As lactate can be used by the liver as an energy substrate, we hypothesized that reduced liver MCT1 expression could promote resistance to hepatic steatosis by altering lipid metabolism though a modulation of AMPK activity.

## 2 Materials and methods

### 2.1 Animals

Experiments were performed in accordance with the Swiss animal welfare laws under the authorization n° VD 2886 from the Service de la consommation et des affaires vétérinaires du Canton de Vaud, Switzerland.

MCT1+/− mice (originally generated by homologous recombination, see [16]) were bred to produce MCT1+/− male mice and MCT1+/+ male littermate controls in the animal facility of the Physiology department at the University of Lausanne, Switzerland. Animals were housed in a controlled environment room, with a temperature of 20–22 °C, relative moisture 50–60%, and 12 h light–dark cycle. At 8 weeks old, MCT1+/− and MCT1+/+ littermates received either a standard diet (SD) (Provimi Kliba, Penthalaz, Switzerland; Cat no 3336) or a high fat diet (HFD) (Harlan Teklad, Oxon, UK; Cat no TD.93075) with unlimited access to food and water during 12 weeks. At the end of the 12 weeks diet period, animals were anesthetized with an intraperitoneal injection of sodium pentobarbital (50 mg/kg), and tissues were collected weighted and frozen in liquid nitrogen for further analysis. Blood glucose, ketone bodies, and lactate were measured from tail vein using specific apparatus (Free Style precision, Abbott, Oxon, UK for ketone bodies and glucose; The Edge analyzer, Apex Biotechnology Corp., Taiwan for lactate).

### 2.2 Total RNA isolation and real-time quantitative PCR analysis

Total RNA was extracted from frozen liver using the Prep Ease RNA/Protein Spin Kit Affymetrix (High Wycombe, United-Kingdom) according to the manufacturer's protocol. RNA was reverse-transcribed into cDNA with the use of Takara RT-kit (Takara Biotechnology, Saint-Germain-en-Laye, France). The abundance of transcripts was assessed by real-time PCR (ViiA7 Real time PCR system life Technologies, Zug, Switzerland) with a SYBR Green detection system (Applied Biosystem, Rotkreuz, Switzerland). Differences were calculated using the ΔΔCt method using the Polymerase 2 gene as housekeeping gene (Primers used are shown in Supplementary Table 1).

### 2.3 Western blots

Frozen livers were homogenized in RIPA lysis buffer (Millipore, Zug, Switzerland) with phosphatase and protease inhibitors (Pierce, Lausanne, Switzerland). 30 μg of total proteins were loaded on SDS-PAGE gels and transferred to nitrocellulose membranes. Membranes were blocked with TBS containing 0.1% Tween 20 and 5% BSA, then incubated overnight with primary antibodies (Supplementary Table 2). Blots were revealed by chemiluminescence (WesternBright ECL; Witec ag, Luzern, Switzerland) and imaged with a detection system (Chemidoc XRS+, Biorad, Switzerland). Densitometric analysis of chemiluminescent signals was performed using Image lab software.

### 2.4 Liver lipid measurements

Liver triglycerides were extracted using the method of Bligh and Dyer [22] and measured using a kit from BioMérieux (Genève, Switzerland) according to the manufacturer's instructions.

#### 2.4.1 Liver glycogen content determination

100 mg of tissue were homogenized in citrate buffer (NaF 50 mM, Citric acid 100 mM, pH 4.2) and centrifuged at 5000 g for 10 min at 4 °C. Supernatant was removed, and 460 μl were incubated with 40 μl of a solution of amyloglucosidase 50 U/ml (Sigma) diluted in sodium citrate buffer, while 460 μl were incubated with 40 μl of sodium citrate buffer only. Tubes were shaken for 30 min at 55 °C. Then, 10 μl of each sample were deposited in 96 well plates with 200 μl of a RTU (Ready to Use) buffer (BioMérieux), incubated at room temperature for 20 min. The optical density was measured at 505 nm by spectrophotometry. The difference between conditions with amyloglucosidase or buffer only represents the glycogen content as mg of glucose from glycogen hydrolysis per g of liver.

### 2.5 Hyperinsulinemic-euglycemic clamp

After 12 weeks of diet, mice were anesthetized with 50 mg/kg sodium pentobarbital (i.p.). An indwelling catheter was introduced in the left jugular vein and externalized on the back. Diet was restricted to 6 h, and mice underwent a euglycemic clamp with a continuous infusion of human insulin (Novorapid, Novo Nordisk) at a rate of 8 mU/kg/min. Glucose (15%) was infused at variable rates to maintain euglycemia. Insulin-stimulated whole-body glucose flux was estimated using a continuous infusion of [3-^3^H] glucose at a rate of 0.09 μCi/min throughout the clamp procedure. Blood samples (10 μl) were collected from the tail vein for plasma [3-^3^H] glucose determination at 10-min intervals during the last 30 min. Rates of whole-body glucose disappearance (Rd), Hepatic glucose production (HGP) and Glucose infusion rate (GIR) were calculated as previously described [23]. Additional blood samples were collected to measure plasma insulin concentrations before and at the end of the clamp using ELISA specific kits from Merk Millipore according to manufacturer's instructions.

### 2.6 Pyruvate and lactate tolerance tests

6 h fasted mice received an intraperitoneal pyruvate (2 mg/g) or lactate (1 mg/g) injection. Blood was collected from the tail vein at – 30, 0, 15, 30, 45, 60, 90, and 120 min for determination of glucose or lactate levels using specific apparatus (Free Style precision, Abbott, Oxon, UK for glucose; The Edge analyzer, Apex Biotechnology Corp., Taiwan for lactate).

### 2.7 Histological analysis

Animals were anesthetized and killed by cervical dislocation. An incision in the skin was made from the rectum to the esophagus and the entire animal was put in a buffered formol fixating solution for 24 h. Liver was dissected, paraffin-embedded with a Leica ASP300S tissue processor (Leica, Heerbrug, Switzerland), and 3 μm tissue sections prepared with a Microm HM 335 E microtome (Thermo Scientific, Walldorf, Germany). Each section was routinely stained with hematoxylin and eosin, mounted on glass slides, and examined with a Nikon Eclipse 80i microscope (Nikon AG, Egg, Switzerland) using brightfield optics at 20× and 40× magnification. On digital images of representative sections the surface of lipid droplets was measured and the ratio to the total tissue surface was calculated.

### 2.8 Statistical analysis

Results are presented as mean ± SEM. Statistical analysis was performed using Prism 6.01. Normality was tested with the Kolmogorov–Smirnov test. For each experiment, a one or two-way ANOVA test was performed. Significant differences are indicated as * for a significant effect of HFD in WT and HE mice; § for a significant effect of the transgene under SD; and # for a significant effect of the transgene under HFD.

## 3 Results

### 3.1 MCT1+/− mice are protected from HFD-induced insulin resistance

MCT1 haploinsufficient (MCT1+/−) mice are resistant to the development of obesity and the appearance of hepatic steatosis when fed a high fat diet as well as to the development of chronic hepatic inflammation as indicated by the absence of TNFα mRNA induction under HFD (Supplementary Figure 1A–E). In addition, in accordance with glycemia measurements (Supplementary Figure 1H), plasma insulin levels were increased in MCT1+/+ mice under HFD, but not in MCT1+/− mice (Figure 1A). Using a hyperinsulinemic euglycemic clamp, insulin resistance in MCT1+/+ mice was revealed after 12 weeks of HFD as shown by both decreased glucose infusion rate (GIR) (Figure 1B) and decreased rate of whole-body glucose disappearance (Rd) (Figure 1C). In contrast, MCT1+/− mice showed no sign of insulin resistance based on these two parameters when fed with either a SD or HFD (Figure 1B–C). The calculated hepatic glucose production (HGP) during hyperinsulinemia was not altered in both MCT1+/+ and MCT1+/− mice (Figure 1D).

**Figure 1:**
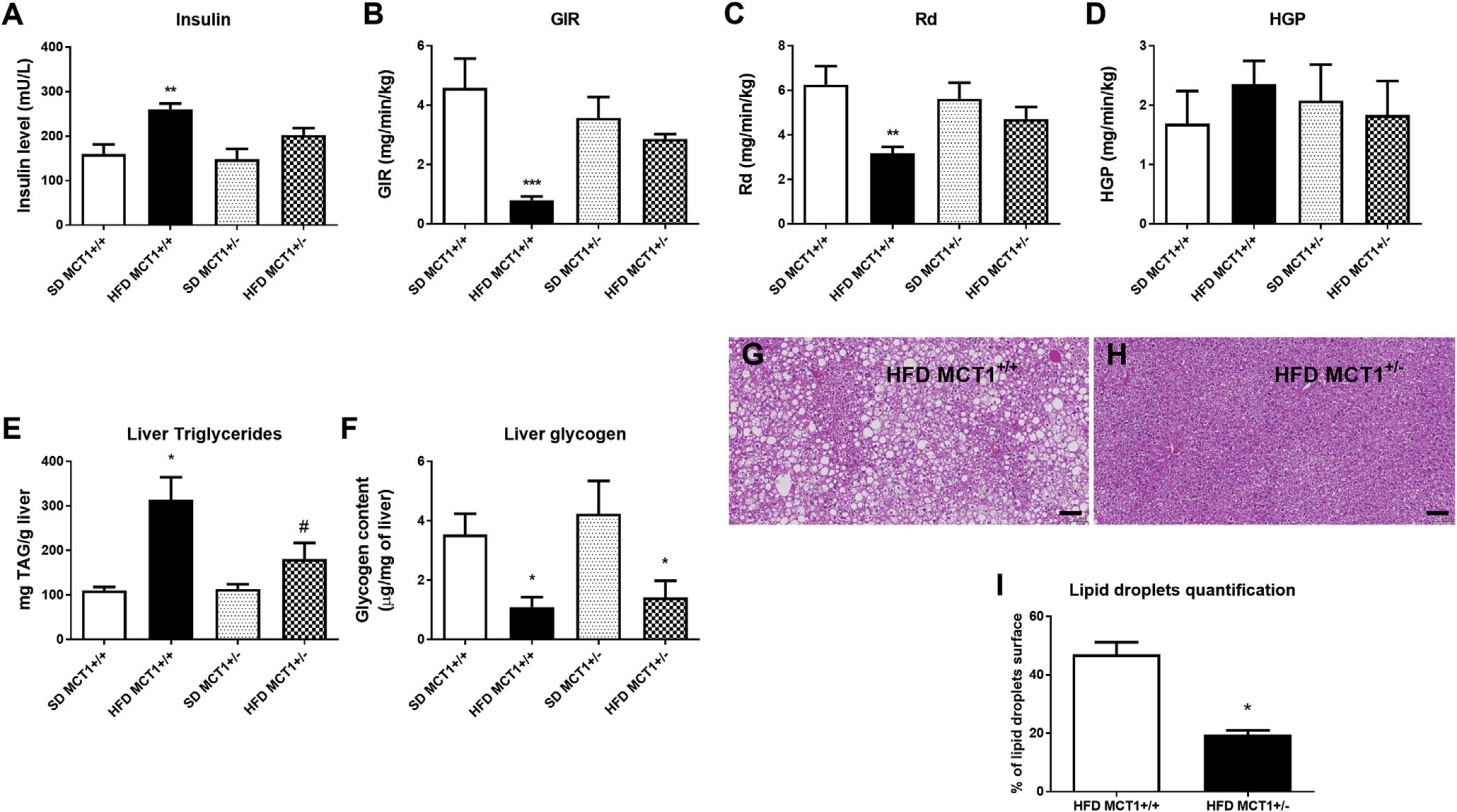
**MCT1+/− mice are resistant to HFD-induced obesity and insulin resistance, accumulate less triglycerides and have a decreased glycogen liver content.** (A) Blood insulin level measured at the end of the 12 weeks HFD that induced hyperinsulinemia in MCT1+/+ mice but not in MCT1+/−. (B–D) Hyperinsulinemic-euglycemic clamps show insulin resistance in MCT1+/+ mice under HFD but not in MCT1+/−. Results presented show Glucose infusion Rate (GIR) to maintain euglycemia (B), Rd represents the glucose disappearance rate (C) and HGP represents Hepatic Glucose production (D). (E) Liver triglycerides content increased in MCT1+/+ mice after HFD, whereas in MCT1+/− mice it did not. (F) 12 weeks HFD induced a decrease in glycogen content in both MCT1+/+ and MCT1+/− mice. n = 12 for each group. (G–H) Comparative histological analysis of liver from MCT1+/+ and MCT1+/− mice under HFD stained with hematoxylin/eosin and examined under light microscopy at 40× magnification. Calibration bar, 100 μm. (I) Relative quantification of lipid droplets surface ratio compared to total surface. n = 2 for each group. *represents differences due to diet, # represents differences between genotypes during HFD. * or #: p < 0.05; ** or ##: p < 0.01.

### 3.2 MCT1+/− mice are resistant to HFD-induced hepatic lipid accumulation

To determine how these MCT1+/− mice resist to hepatic steatosis, hepatic lipid content was quantified. An increased triglyceride liver content was observed in MCT1+/+ mice after HFD, while it was significantly less in MCT1+/− mice (Figure 1E). Interestingly, liver glycogen content was decreased after HFD in both MCT1+/+ and MCT1+/− mice, suggesting similar glycogen metabolism in both groups (Figure 1F). Histological analysis of the liver revealed an important hepatic steatosis in MCT1+/+ mice fed HFD while it was strongly reduced in the liver of MCT1+/-mice fed HFD (Figure 1G–H). Quantitatively, lipid droplets accounted for 51% of the surface in the liver of MCT1+/+ mice fed HFD while it represented only 20% in the liver of MCT1+/− mice (Figure 1I).

### 3.3 MCT1+/− mice under HFD exhibit decreased responses in both pyruvate and lactate tolerance tests

Glucose production was assessed first with a pyruvate tolerance test. MCT1+/+ mice under HFD showed a higher glucose production rate than MCT1+/+ mice fed SD which remained elevated at the end of the test period. In contrast, MCT1+/− mice under HFD displayed a transient elevation of glycemia induced by pyruvate which normalized 60 min after pyruvate injection as observed for SD fed mice as shown by the increased area under the curve (a.u.c) (Figure 2A,B), indicating increased liver glucose production. Since MCT1 is a lactate transporter which could be essential for neoglucogenesis, a lactate tolerance test was performed. This test revealed a similar response than the one with pyruvate for MCT1+/+ mice under HFD with a long-lasting increase of glycemia compared to SD fed mice. MCT1+/− mice under HFD did not produce such a response upon lactate injection. Interestingly, under SD, even MCT1+/− mice presented a lower response than MCT1+/+ mice (Figure 2C,D). Blood lactate levels were measured after lactate injection. Lactate levels first increased after infusion and then normalized in SD fed groups but not completely in HFD fed groups. Thus, HFD fed mice (either MCT1+/+ or +/−) exhibited long-lasting elevation of blood lactate levels in response to a lactate injection (Figure 2E,F). In order to understand the cause of these anomalous responses to pyruvate and lactate, hepatic expression of key enzymes for their metabolism was determined. Expression of pyruvate carboxylase (which converts pyruvate into oxaloacetate) was induced only in HFD fed MCT1+/+ mice (Figure 2G). Expression of the lactate dehydrogenase isoform LDHA, which preferentially converts pyruvate into lactate, was not altered either by the transgene nor the diet (Figure 2H). In contrast, under HFD, MCT1+/+ mice exhibited an increase in the expression of the LDHB isoform which is preferentially involved in the conversion of lactate into pyruvate. Interestingly, this effect on LDHB was not detected in MCT1+/− mice after HFD (Figure 2I).

**Figure 2:**
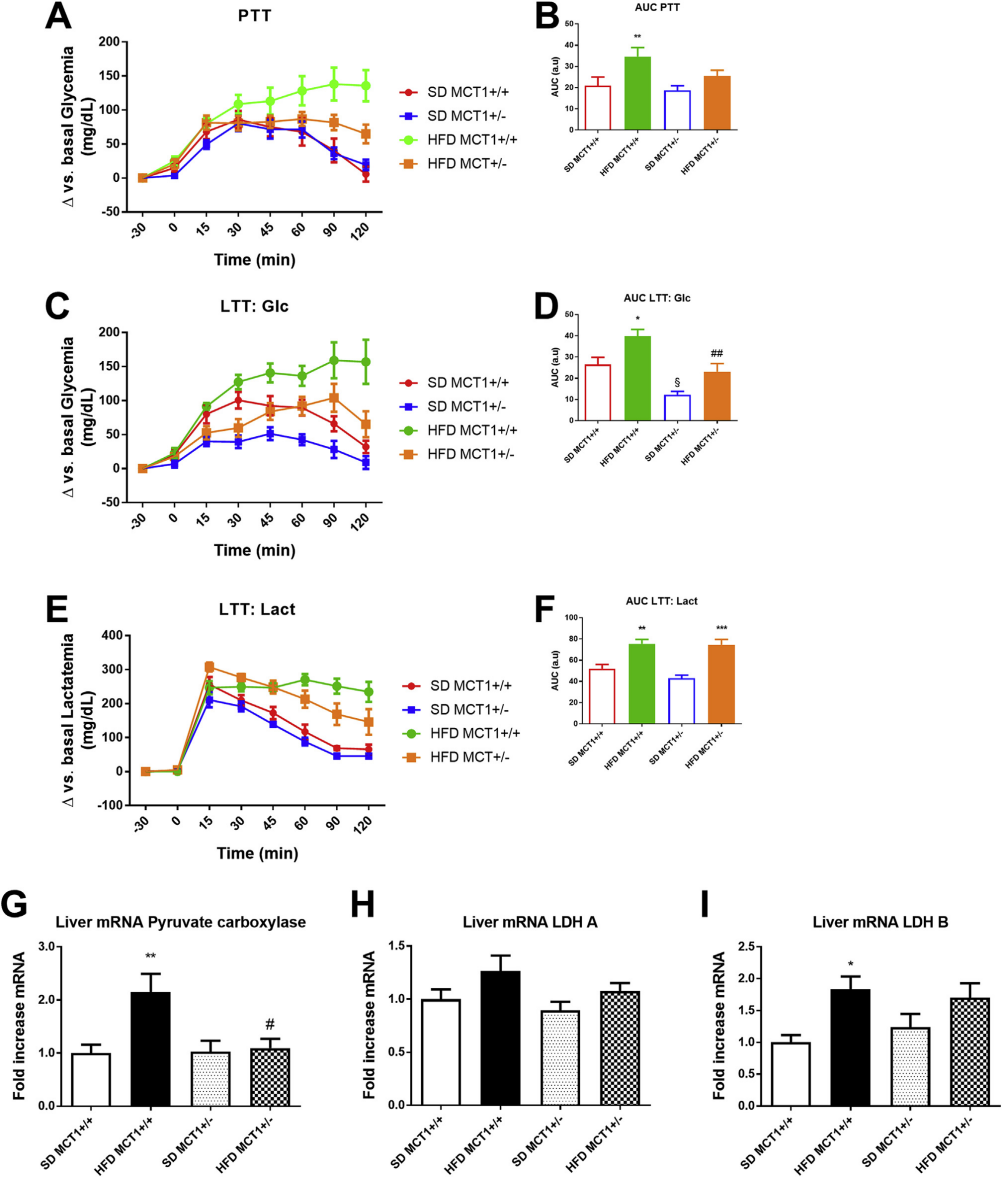
**HFD causes an increase in glucose production following pyruvate or lactate injection in MCT1+/+ mice but not in MCT1+/− mice.** (A) Blood glucose level measurement following a pyruvate injection in MCT1+/+ and MCT1+/− mice at 12 weeks of SD or HFD and (B) Area under the curve. (C) Blood glucose level measurement following a lactate injection in MCT1+/+ and MCT1+/− mice at 12 weeks of SD or HFD and (D) Area under the curve. (E) Blood lactate level measurement following a lactate injection in MCT1+/+ and MCT1+/− mice at 12 weeks of SD or HFD and (F) Area under the curve. After 12 weeks of SD or HFD, liver mRNA expression levels for (G) Pyruvate carboxylase (H) Lactate dehydrogenase A (LDH A) and (I) Lactate dehydrogenase B (LDH B) isoforms. n = 12 for each group. * represents differences due to diet, # represents differences between genotypes during HFD. * or #: p < 0.05; ** or ##: p < 0.01; *** or ###: p < 0.001.

### 3.4 Mitochondrial complexes content is enhanced in MCT1+/+ mice but not in MCT1+/− mice under HFD

**Figure 3:**
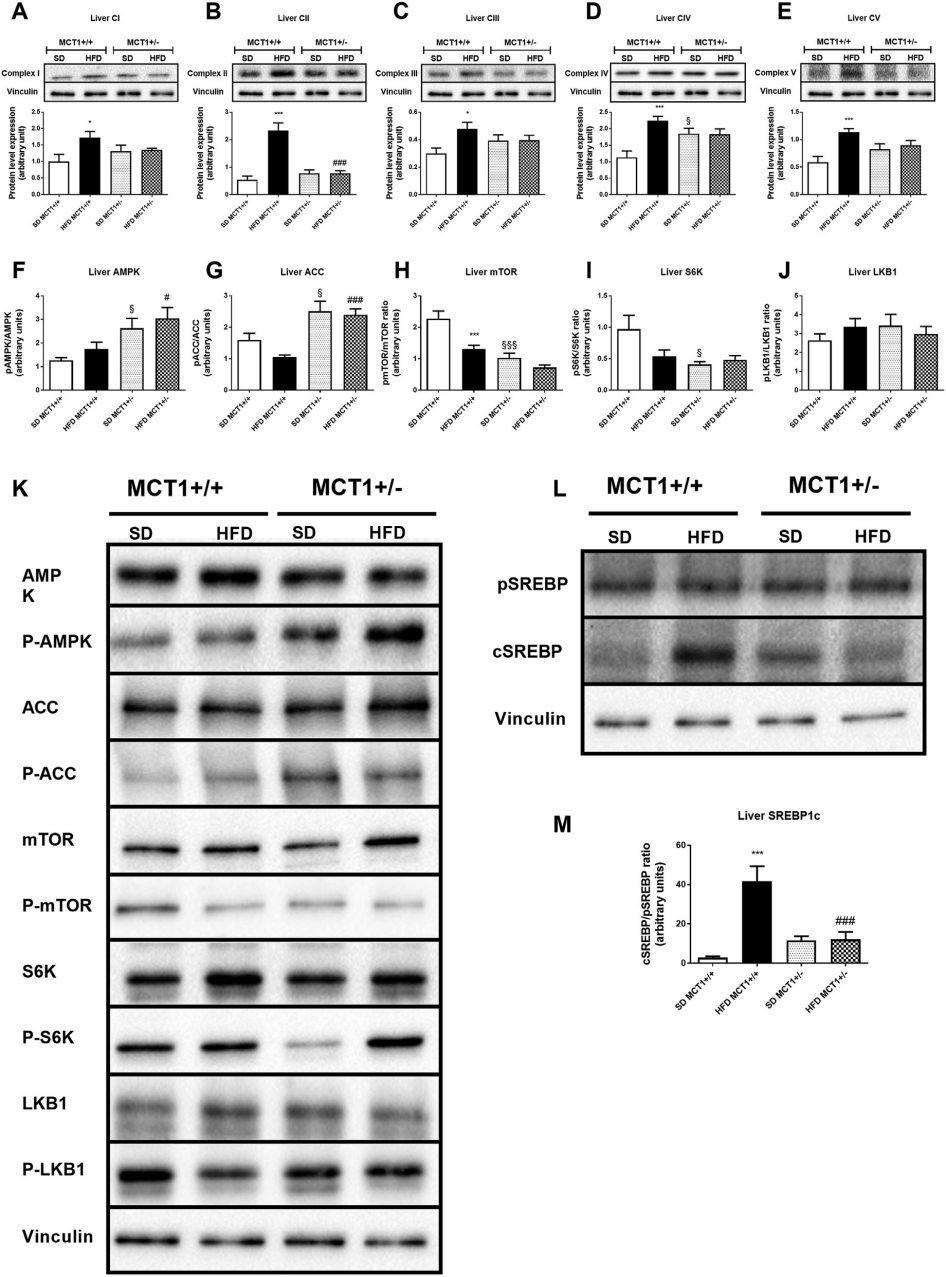
**Resistance to HFD-induced obesity in MCT1+/− mice is associated with AMPK activation independently of LKB1 activity but correlated with lower oxidative phosphorylation capacities leading to SREBP1 inhibition.** (A–E) Upper panels are representative western blots for OXPHOS complexes protein level. Lower panel provides the quantification of protein expression (F–J) Ratio of phosphorylated to non-phosphorylated isoform protein for AMPK (F), ACC (G), mTOR (H), S6K (I), and LKB1 (J) and (K) representative western blots for the same proteins and phosphorylation forms. (L) Representative western blot for precursor and cleaved forms of SREBP1. (M) Quantification of Cleaved to precursor SREBP1 isoforms ratio. n = 7 animals per condition. *represents differences due to diet, § represents differences between genotypes during SD, and # represents differences between genotypes during HFD. *, § or #: p < 0.05; **, §§, or ## indicates p < 0.01; ***, §§§, or ###: p < 0.001.

Our aforementioned results suggest an alteration in oxidative metabolism. Indeed, overexpression of LDHB could promote enhanced pyruvate production from lactate. In turn, increased pyruvate carboxylase expression suggests a higher activity of the Krebs cycle and of oxidative phosphorylation. Expression levels of the four mitochondrial complexes composing the respiratory chain were increased in HFD fed MCT1+/+ mice (Figure 3A–D). Moreover, expression of ATP synthase (or Complex V) was also increased in MCT1+/+ mice fed a HFD, suggesting an increased ATP production capacity (Figure 3E). Interestingly, MCT1+/− mice did not undergo such modifications in expression following HFD. However, expression of Complex IV was higher compared to MCT1+/+ mice under SD (Figure 3D). This result suggests that the oxidative potential could be constitutively higher in the MCT1+/− mouse model under SD but not under HFD.

### 3.5 AMPK is constitutively activated in MCT1+/− mice

Given that modifications in oxidative metabolism should have an impact on ATP production, we postulated that the energy status-sensitive enzyme AMPK could be affected. No modification in the phosphorylated state of AMPK was detected in MCT1+/+ mice after HFD (Figure 3F,K). However, a higher level of AMPK phosphorylation was seen in MCT1+/− mice compared to MCT1+/+ mice under SD, indicating a constitutively higher AMPK activity. Under HFD, this increased activation was still present (Figure 3F,K). In accordance with this activation, the AMPK target enzyme Acetyl-CoA Carboxylase (ACC) was also phosphorylated and thus activated in MCT1+/− mice, whereas there was no significant modification in MCT1+/+ mice (Figure 3G,K). Surprisingly, mTOR (putatively in both mTORc1 and c2 complexes) and S6K, two known downstream targets of AMPK signaling, exhibited a decreased phosphorylation ratio in MCT1+/− mice (Figure 3H,I, and K). mTOR activation was also decreased in MCT1+/+ mice fed with HFD, indicating that another regulating pathway than AMPK should be involved in mTOR signaling in this condition. In addition, the upstream enzyme in AMPK signaling, LKB1, known to activate AMPK, did not present any modification of its phosphorylation state, and thus of its activation (Figure 3J,K). Such a result indicates that AMPK activation did not result from LKB1 signaling.

### 3.6 SREBP1 precursor cleavage is prevented in MCT1+/− mice under HFD and could protect MCT1+/− mice against hepatic steatosis

Recently, resistance to hepatic steatosis was associated with an inhibition of the production of the mature form of SREBP1 via activated AMPK [19]. To assess this possibility, the cleaved/precursor ratio of SREBP1 was determined by western blot. We observed an increased ratio in HFD fed MCT1+/+ mice due to an increased appearance of the cleaved form (Figure 3L and M). This cleaved form did not increase in MCT1+/− mice either under SD or HFD condition. This suggests that constitutively active AMPK might phosphorylate SREBP1 and prevent its active form to be generated in MCT1+/− mice [19].

### 3.7 Expression of key hepatic genes revealed a decrease in lipid metabolism in MCT1+/− mice

**Figure 4:**
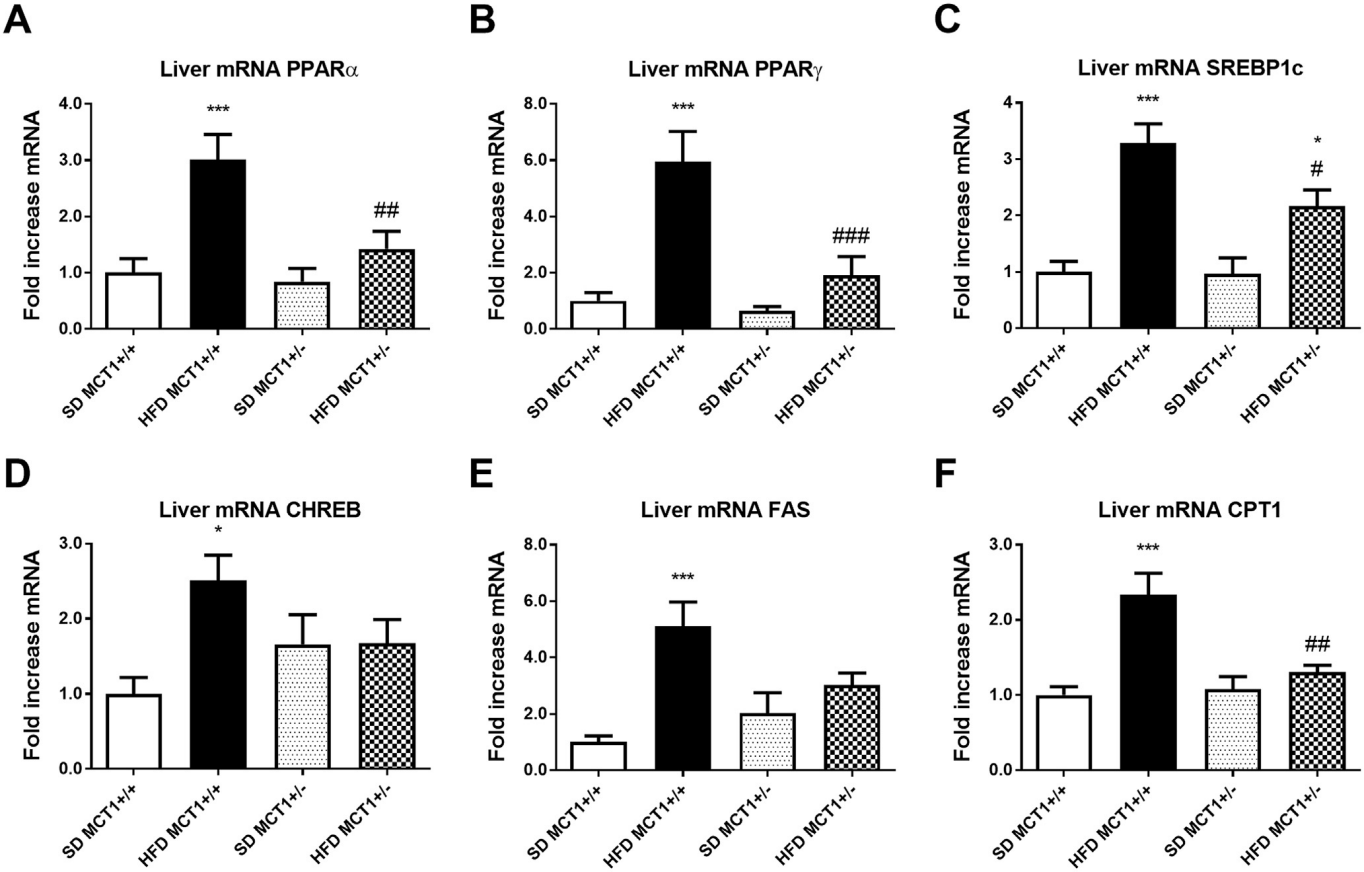
**HFD causes an increase in mRNA levels of genes involved in lipid metabolism and a metabolic shift increasing lipid metabolism in MCT1+/+ but not in MCT1+/− mice.** After 12 weeks of SD or HFD, liver mRNA expression levels for transcription factors PPARα and PPARγ, SREBP1, CHREB (A–D), for the enzyme involved in lipid metabolism FAS (E) or the mitochondrial lipid transporter CPT1 (F). n = 10 animals per condition. *represents differences due to diet, # represents differences between genotypes during HFD. * or #: p < 0.05; ** or ##: p < 0.01; *** or ###: p < 0.001.

Gene expression analysis for specific proteins involved in lipid metabolism such as the transcription factors PPARα and γ showed a robust increase in expression following HFD in MCT1+/+ mice but not in MCT1+/− mice (Figure 4A,B). In accordance with previous studies, HFD also enhanced the mRNA expression levels of SREBP1 and CHREBP, both being involved in lipid and carbohydrate metabolism (Figure 4C,D). Interestingly, SREBP1 was induced by HFD in both MCT1+/+ and MCT1+/− mice. However, the expression level after HFD was significantly lower in MCT1+/− mice than in MCT1+/+ mice (Figure 4C). Expression of Fatty Acid Synthase (FAS) was increased in MCT1+/+ mice following HFD but not in MCT1+/− mice (Figure 4E). Finally, expression of the lipid transporter CPT1 was induced in MCT1+/+ mice fed a HFD, indicating an increase in fat oxidation in mitochondria, but this was not the case in HFD fed MCT1+/− mice (Figure 4F).

## 4 Discussion

In the present study, we report a new signaling pathway putatively implicated in hepatic steatosis development during HFD-induced obesity involving lactate metabolism and AMPK activity. Hepatic steatosis is known to result from a cascade of events leading to lipid accumulation in the liver [1], [14]. High fat diet-induced obesity is classically used as an animal model of hepatic steatosis. A recent mouse model resistant to HFD-induced obesity and hepatic steatosis was generated to better understand the development of such a disease [16]. This mouse model presents the particularity to be haploinsufficient for the monocarboxylate transporter MCT1. Monocarboxylates, which include lactate, pyruvate, and ketone bodies, have been poorly studied in the context of obesity development in the past years. Nevertheless, different groups have highlighted a putative implication of these substrates in metabolic control and dysregulations but a clear role remains to be demonstrated [18].

A histological analysis (and liver TG measurements) in MCT1+/− mice under HFD revealed a decreased lipid accumulation in the liver, supporting a resistance to hepatic steatosis. This decreased lipid accumulation most likely results from a decrease in lipid metabolism in this mouse model under HFD as suggested by the reduced gene expression of key factors involved in lipid metabolism. Usually, hepatic steatosis is associated with insulin resistance. Here, by using the hyperinsulinemic-euglycemic clamp technique, we demonstrated that MCT1+/− mice did not develop insulin resistance after 12 weeks of HFD. However, hepatic glucose production was similar during the clamp between groups, even in MCT1+/+ mice under HFD, suggesting that insulin resistance mainly affects other tissues than liver such as adipose tissue and/or muscle. In our conditions, insulin resistance did not affect hepatic glucose production but it could still impact liver lipid metabolism [24], [25].

MCT1 transports lactate, pyruvate and ketone bodies in various tissues [17]. Ketone bodies are metabolites produced by lipid oxidation essentially in the liver but used as energy substrates by muscles, the heart and the brain. In contrast, lactate and pyruvate are related to glucose metabolism and are exchanged between various organs and tissues. It has been shown that ketone bodies are secreted by hepatocytes via a different monocarboxylate transporter than MCT1 [26]. Thus, the role of MCT1 in the liver might be predominantly related to lactate metabolism. Indeed, pyruvate injection produced a similar response in MCT1+/+ and MCT1+/− mice under SD, indicating a metabolism not affected by the haploinsufficiency. In contrast, MCT1+/− mice present a lower response to lactate for glucose production but a normal response in terms of blood lactate. Such results suggest that lactate metabolism *per se* is altered in hepatocytes. Interestingly, hepatocytes also express MCT2, a monocarboxylate transporter with a higher affinity for pyruvate although it also transports lactate [27]. We found that the expression of MCT2 mRNA is enhanced in the liver of MCT1+/+ under HFD while this effect was blunted in MCT1+/− mice (see Supplementary Figure 1I). This differential response of MCT2 expression in liver, combined with the reduction in hepatic MCT1 expression for MCT1+/− mice, can explain the hepatic metabolic shift observed between MCT1+/+ and MCT+/- mice in absence of any change in lactatemia.

Gene expression analysis revealed that HFD feeding was associated with a shift in the ratio of LDH isoforms, with an increase in LDHB. If LDHA preferentially converts pyruvate into lactate, the LDHB isoform is associated with pyruvate formation from lactate. Thus, these results point to a possible role of circulating lactate in sustaining hepatic ATP production by providing pyruvate as an oxidative substrate. This effect is permitted in MCT1+/+ mice by the increased expression of the MCT2 transporter while MCT1+/− mice did not display such an effect (Supplementary Figure 1I). Moreover, lactate taken up by hepatocytes can be converted to pyruvate that will contribute to the increased neoglucogenesis observed with the pyruvate tolerance test and thus participate to elevate glycemia in MCT1+/+ mice under HFD [30]. MCT1+/− mice do not show increased hepatic LDHB isoform expression. Interestingly, LDHB induction in muscle was recently correlated with a decrease in pH associated with an increase in intracellular lactate concentration [28]. Thus, an increase in lactate uptake via both MCT1 and MCT2 in hepatocytes with a lowering of intracellular pH could be a trigger for enhanced LDHB expression as observed in muscle [28]. In our transgenic mouse model, the reduced hepatic MCT1 transporter expression (as well as the lack of enhanced MCT2 expression) could prevent the pH/lactate-dependent induction in LDHB isoform in hepatocytes and the elevated production of pyruvate. Subsequently, it would lead to a decreased rate of neoglucogenesis as well as a reduction of the Krebs cycle activity by providing lower amounts of acetyl-CoA and oxaloacetate via pyruvate carboxylase. In short, under HFD, oxidative phosphorylation should be less active in MCT1+/− mice compared to MCT1+/+ mice. This is supported by the lack of enhancement in expression levels of mitochondrial complexes in MCT1+/− mice under HFD compared to MCT1+/+ mice. As a consequence, it is postulated that the reduced hepatic ATP production would increase the AMP/ATP ratio leading to the increased activation of AMPK. Interestingly, analysis of some signaling pathways downstream of AMPK show that they are not activated. Furthermore, the upstream activator of AMPK, LKB1, is not activated either. Thus, the decreased pyruvate synthesis in MCT1+/− mice and the subsequent decrease in ATP production resulting in a rise of the AMP/ATP ratio is the most likely cause of the active AMPK in MCT1+/− mice. The consequence of this AMPK activation is the phosphorylation of a limited and specific set of downstream targets of AMPK such as ACC. Phosphorylation of ACC will reduce the production of malonyl-CoA, a known inhibitor of fatty acid oxidation [29].

AMPK activation was recently shown to phosphorylate at a specific site and inhibit SREBP1 [19]. This AMPK-dependent phosphorylation was demonstrated to protect against hepatic steatosis by inhibiting lipid metabolism and lipid accumulation. In MCT1+/+ mice fed HFD, an increase in the mature, activated form of SREBP1 could be detected. This is consistent with a low phosphorylation level of SREBP1 at the AMPK target site. In contrast, MCT1+/− mice present a decreased level of the active form of SREBP1. Thus, it appears that activated AMPK indeed inhibits SREBP1 in MCT1+/− mice. As a consequence, several key enzymes of lipid metabolism are not induced by the exposure to a high fat diet in hepatocytes, leading to a protection against lipid accumulation and hepatic steatosis in these mice.

## 5 Conclusion

To summarize, the MCT1 haploinsufficient mouse model allowed us to unravel a key role of lactate metabolism in steatosis onset. In wildtype mice, the expression of the LDHB isoform is elevated in the liver, most likely as a consequence of lactate uptake and intracellular acidification. In turn, LDHB leads to elevated pyruvate formation from lactate, contributing to both neoglucogenesis and enhanced ATP synthesis, the former by stimulating oxidative phosphorylation. High ATP production capacity results in a decrease of AMPK phosphorylation (and thus activity), leading to increased SREBP1 activity that induces different pathways causing lipid accumulation in the liver. In contrast, in MCT1 haploinsufficient mice, the reduction in lactate uptake caused by lower MCT1 (and MCT2) expression levels in hepatocytes prevents under HFD the high LDHB expression and the subsequent cascade. Thus, pyruvate synthesis is reduced and AMPK becomes more active due to a reduced ATP production via oxidative phosphorylation. Consequently, SREBP1 is inhibited, preventing the activation of lipid metabolism and leading to protection against hepatic steatosis. In conclusion, our results reinforce the concept that AMPK activation promotes a protection against hepatic steatosis by inhibiting SREBP1. It also reveals an unexpected regulatory role of circulating lactate in the regulation of hepatic lipid metabolism (Supplementary Figure 2). Nonetheless, we do not exclude that other tissues or organs implicated in the metabolic dysfunctions associated with obesity, e.g. adipose tissue (which also express MCT1), could be affected and contribute to the protection against hepatic steatosis observed in MCT1 haploinsufficient mice.

## Author contributions

L.C. designed and performed experiments, developed the hypothesis, analyzed data, prepared figures, and wrote the manuscript. M.A., C.S., J.S., and C.L. performed experiments and analyzed data. C.R. participated in experiments. F.R.J. and L.P. supervised the work as well as wrote the manuscript with input from all authors.

## Funding

This study was supported by grants from the “Société francophone du Diabète” in 2014 and 2016 attributed to LC. LP received financial support for this project from the Department of Physiology, University of Lausanne and from the program IdEx Bordeaux ANR-10-IDEX-03-02. FRJ was supported by a grant from the Leenaards Foundation and the Raymond Berger Foundation, Lausanne.

## Figures and Tables

**figs1:**
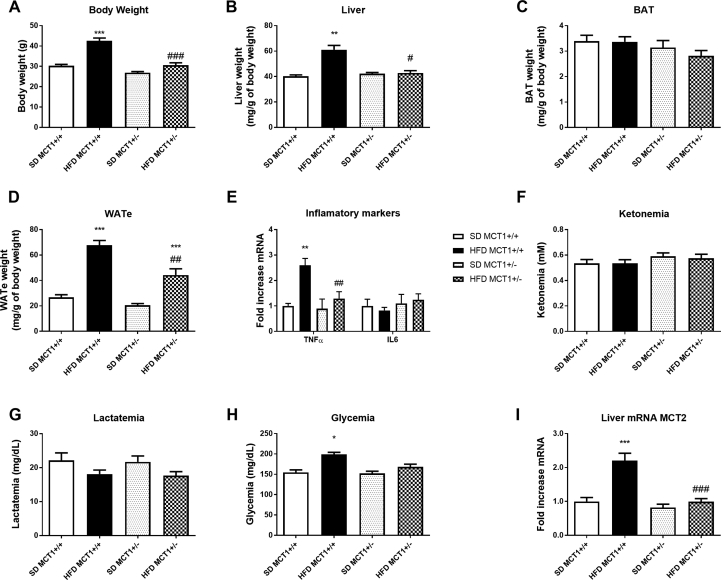


**figs2:**
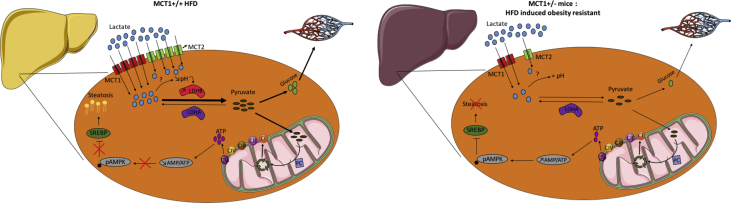


## Appendix A Supplementary data

mmc1 mmc1
