# Supplementary material for: AMPK activation caused by reduced liver lactate metabolism protects against hepatic steatosis in MCT1 haploinsufficient mice
Source: Mol Metab. 2017 Oct 20;6(12):1625–33. doi: 10.1016/j.molmet.2017.10.005 (PMC5699913; doi:10.1016/j.molmet.2017.10.005)
Supplement: mmc1 [file mmc1.docx]

**SUPPLEMENTARY METHODS**

At the end of the 12 weeks diet period, animals were anesthetized with an intraperitoneal injection of sodium pentobarbital (50mg/kg) and tissues were collected weighted and frozen in liquid nitrogen for further analysis. Blood glucose, ketone bodies and lactate were measured from tail vein using specific apparatus (Free Style precision, Abbott, Oxon, UK for ketone bodies and glucose; The Edge analyzer, Apex Biotechnology Corp., Taiwan for lactate).

**Supplementary Figure 1: MCT1+/- mice present a HFD induced obesity resistance with unaltered body weight or tissue weights associated with normal blood circulating glucose and monocarboxylates levels.** (A) Body weight of WT MCT1+/+ mice and MCT1 deleted MCT1+/- mice fed a standard diet (SD) or a high fat diet (HFD) during 12 weeks. (B-D) Liver, Brown Adipose Tissue (BAT) and epididymal White Adipose Tissue fat pad (WATe) weighted at the end of the 12weeks diet period. (G) After 12 weeks of SD or HFD, liver mRNA expression levels coding for TNFα and IL6. (F-H) Ketone bodies (E), Lactate (F) and Glucose (G) blood levels at the end of the 12weeks diet period. n=20 for each group.

* represents differences due to diet, # represents differences between genotypes during HFD. * or #: p<0.05; ** or ## : p<0.01; *** or ### : p<0.001.

**Supplementary Figure 2: Schematic representation of the proposed mechanism involved in MCT1+/- mouse resistance to hepatic steatosis development.** WT mice fed with HFD show increased MCT2 levels leading to increased lactate uptake in hepatocytes that induces a decrease in pH. Such decreased pH in turn leads to an overexpression of LDH B increasing the rate of pyruvate formation from lactate. This pyruvate is used in the neoglucogenic pathway, participating in the hyperglycemia observed. In parallel, part of pyruvate sustains oxidative phosphorylation through Pyruvate carboxylase (PC) activity which increases the ATP production. This ATP will then decrease the ratio AMP/ATP, preventing the activation of AMPK. Thus, inactivated AMPK fails to inhibit the SREBP1c transcription factor that will stimulate lipid metabolism and lipid accumulation in hepatocytes participating in the development of steatosis. In MCT1+/- mice deleted for the lactate transporter MCT1, it is postulated that the decreased lactate uptake also due to the absence of an increase in MCT2 expression will prevent LDH B expression. Thus, the LDH A isoform mainly converts pyruvate in lactate reducing pyruvate utilization for glucose production and for oxidative phosphorylation. Since respiration decreases, ATP production falls and increases the ratio AMP/ATP that promotes AMPK activity. Activated AMPK will then inhibit SREBP1c which is shown to protect against hepatic steatosis [19].

**Supplementary Table 1: Primers sequences list**

| **Gene** | **Forward** | **Reverse** |
| --- | --- | --- |
| ATGL | GTC CTT CAC CAT CCG CTT GTT | CTC TTG GCC CTC ATC ACC AG |
| BHBDH | TGC AAC AGT GAA GAG GTG GAG AAG | CAA ACG TTG AGA TGC CTG CGT TGT |
| CHREB | CAA GTT GCT ATG CCG GGA CAA | CCT CCG TTG CAC ATA CTG ATT |
| CPT1 | ACC CCA GTG CCT TAA CGA TG | GAA CTG GTC GCC AAT GAG AT |
| DGAT2 | TGG GCC TTG GTG GTT TCT TAC | GAC TGC CCT TGC CCA GCT A |
| FAS | CAT GAC CTC GTG ATG AAC GTG T | CGG GTG AGG ACG TTT ACA AAG |
| GLUT2 | TTC CGG AAG AAG AGT GGT TCG | TGG TCG GTT CCT CGG TTT TAG |
| HMGcs2 | TGG TTC AAG ACA GGG ACA CAG AAC | AGA GGA ATA CCA GGG CCC AAC AAT |
| IL6 | CCT CTG GTC TTC TGG AGT ACC | ACT CCT TCT GTG ACT CCA GC |
| PPARa | GCA GCT CGT ACA GGT CAT CA | CTC TTC ATC CCC AAG GCT AG |
| PPARg | TCG CTG ATG CAC TGC CTA TG | GAG AGG TCC ACA GAG CTG ATT |
| PyrCarb | CAT ATG TGG CCC AGT GGT AGA ACA | GGA GAA GCC ATA ACT TGG GTC TGT |
| SREBP1 | CAG CTC AGA GCC GTG GTG A | TTG ATA GAA GAC CGG TAG CGC |
| UCP2 | ATG GTT GGT TTC AAG GCC ACA | TTG GCG GTA TCC AGA GGG AA |
| LDHA | CAA AGT CCA AGA TGG CAA CCC | AGC ACC AAC CCC AAC AAC TGT |
| LDHB | ATT GCG TCC GTT GCA GAT G | TCC CAG AAT GCT GAT GGC A |
| TNFα | ATG AGC ACA GAA AGC ATG A | AGT AGA CAG AAG AGC GTG GT |
| Polymerase2 | CCC TCA TCA TAC CTG GAC ACA TC | GTA AGG GCC ACT ATC TTC ATC ATC |

**Supplementary Table 2: Antibody list**

| **Protein** | **Supplier** | **Dilution** | **Secondary antibody and dilution** |
| --- | --- | --- | --- |
| OXPHOS | Abnova | 1:500 | Mouse, 1:5000 |
| AMPK | Cell Signaling | 1:1000 | Rabbit, 1:10000 |
| P-AMPK | Cell Signaling | 1:1000 | Rabbit, 1:10000 |
| ACC | Cell Signaling | 1:1000 | Rabbit, 1:10000 |
| P-ACC | Cell Signaling | 1:1000 | Rabbit, 1:10000 |
| mTOR | Cell Signaling | 1:1000 | Rabbit, 1:10000 |
| P-mTOR (ser2448) | Cell Signaling | 1:1000 | Rabbit, 1:10000 |
| S6K | Cell Signaling | 1:1000 | Rabbit, 1:10000 |
| P-S6K | Cell Signaling | 1:1000 | Rabbit, 1:10000 |
| LKB1 | Cell Signaling | 1:1000 | Rabbit, 1:10000 |
| P-LKB1 | Cell Signaling | 1:1000 | Rabbit, 1:10000 |
| SREBP(cleaved and precursor) | Abcam | 1:1000 | Mouse, 1:5000 |
| Vinculin | Sigma Aldrich | 1:4000 | Mouse, 1:5000 |
